# Nitrogen‐Doped Carbon‐Assisted One‐pot Tandem Reaction for Vinyl Chloride Production via Ethylene Oxychlorination

**DOI:** 10.1002/anie.202006729

**Published:** 2020-09-29

**Authors:** Hongfei Ma, Guoyan Ma, Yanying Qi, Yalan Wang, Qingjun Chen, Kumar R. Rout, Terje Fuglerud, De Chen

**Affiliations:** ^1^ Department of Chemical Engineering Norwegian University of Science and Technology (NTNU) Sem sælands vei 4 7491 Trondheim Norway; ^2^ College of Chemistry and Chemical Engineering Xi'an Shiyou University Xi'an 710065 Shaanxi China; ^3^ Shaanxi Key Laboratory of Carbon Dioxide Sequestration and Enhanced Oil Recovery (under planning) Xi'an 710065 Shaanxi China; ^4^ Sintef Industry Sem sælands vei 2A 7491 Trondheim Norway; ^5^ INOVYN Herøya Industrial Park 3936 Porsgrunn Norway

**Keywords:** carbon, dehydrochlorination, industrial chemistry, oxychlorination, vinyl chloride

## Abstract

A bifunctional catalyst comprising CuCl_2_/Al_2_O_3_ and nitrogen‐doped carbon was developed for an efficient one‐pot ethylene oxychlorination process to produce vinyl chloride monomer (VCM) up to 76 % yield at 250 °C and under ambient pressure, which is higher than the conventional industrial two‐step process (≈50 %) in a single pass. In the second bed, active sites containing N‐functional groups on the metal‐free N‐doped carbon catalyzed both ethylene oxychlorination and ethylene dichloride (EDC) dehydrochlorination under the mild conditions. Benefitting from the bifunctionality of the N‐doped carbon, VCM formation was intensified by the surface Cl*‐looping of EDC dehydrochlorination and ethylene oxychlorination. Both reactions were enhanced by in situ consumption of surface Cl* by oxychlorination, in which Cl* was generated by EDC dehydrochlorination. This work offers a promising alternative pathway to VCM production via ethylene oxychlorination at mild conditions through a single pass reactor.

## Graphical abstract

Vinyl chloride monomer (VCM) is directly produced by a metal‐free nitrogen‐doped carbon catalyst in one step via the ethylene oxychlorination process at 250 °C. Ethylene oxychlorination and high‐temperature dehydrochlorination reactions are integrated into a single pass with remarkable conversion and selectivity. The efficient combination of tandem reactions produces a VCM yield as high as the current two‐step process, and under mild conditions.

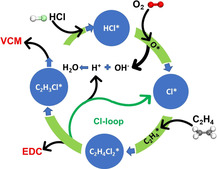

## Introduction

Polyvinyl chloride (PVC) is the most versatile of all thermoplastics that can be used in a wide range of applications. It is the third‐highest volume polymer, slightly behind polyethylene and polypropylene.^[1]^ Vinyl chloride (VCM), the monomer of PVC is the key building block for PVC production, which is mainly produced from ethylene dichloride (EDC) through thermal cracking. Currently, approximately 90 % of VCM production plants worldwide are using a balanced VCM process where Cl_2_ by first chlorinating ethylene to produce EDC, the EDC is then thermally converted to VCM by dehydrochlorination.^[1a]^ The HCl produced in the dehydrochlorination reactor is typically captured and recycled to an oxychlorination reactor to convert C_2_H_4_, O_2_, and HCl to EDC, which is again converted to VCM by dehydrochlorination.[^1a^, ^2^] The balanced VCM process is a complex multi‐process including three reaction sections, separation and recycle unites.^[2]^ Besides, EDC dehydrochlorination also called EDC cracking, is an energy‐intensive process, which is carried out at high temperatures (500–550 °C), high pressures (15–20 bar), at a conversion of approximately 50–60 % leading to an overall VCM yield about 50 % in a single pass.[^1a^, ^3^] The control of impurities and coke formation presented the main challenges in the EDC cracking for the VCM production. The impurities such as butadiene and methyl chloride etc. generated by high‐temperature radicals’ reactions must be controlled at a very low level due to the requirement of plant operation. Coke formation at the high‐temperature cracker tubes is one key parameter influencing the production cost. The complexity of the process drives a search for simplifying the process to produce VCM directly from C_2_H_4_, O_2_, and HCl in a single pass reactor.[^1a^, ^2a^, ^3^]

The ethylene oxychlorination is typically catalyzed by a promoted CuCl_2_/Al_2_O_3_‐based catalyst at relatively low temperatures (ca. 220–250 °C) and 2–6 bar.^[4]^ It is not feasible to integrate the low‐temperature oxychlorination and high‐temperature EDC thermal cracking together, which loses the CuCl_2_/Al_2_O_3_‐based catalyst stability at high temperatures or reduces the activity of EDC dehydrochlorination at low temperatures. Efforts have been devoted to developing new catalysts such as lanthanum (oxy)chloride, and CeO_2_ with bi‐function of oxychlorination and dehydrochlorination for a high‐temperature process to directly produce VCM.[^3^, ^5^] However, these catalysts are not active at low temperatures, the typical ethylene oxychlorination conditions around 220–250 °C. Highly active bifunctional catalysts at low temperatures are highly desired.

In the present work, we report an energy‐efficient process to produce VCM from ethylene oxychlorination at relatively low temperatures on a metal‐free carbon catalyst. To the best of our knowledge, for the first time, a metal‐free carbon‐based catalyst was reported having the catalytic activity for ethylene oxychlorination to directly produce VCM at a relatively low temperature of 250 °C. We found that the N‐doped carbon catalyst not only catalyzes the ethylene oxychlorination but also facilitates the EDC dehydrochlorination reaction due to the bifunctional ability. The oxychlorination–dehydrochlorination process was integrated into one dual‐bed single pass reactor, where ethylene oxychlorination catalysts were followed by N‐doped carbon catalysts, at a low temperature (250 °C) to achieve a high VCM yield up to 76 %, even higher than the one in the current industrial single‐pass balanced VCM process. The one‐pot process has been remarkably intensified by a synergic effect between the oxychlorination and dehydrochlorination reactions at the same type of active site by the surface Cl* looping between the two reactions.

## Results and Discussion

**Figure 1:**
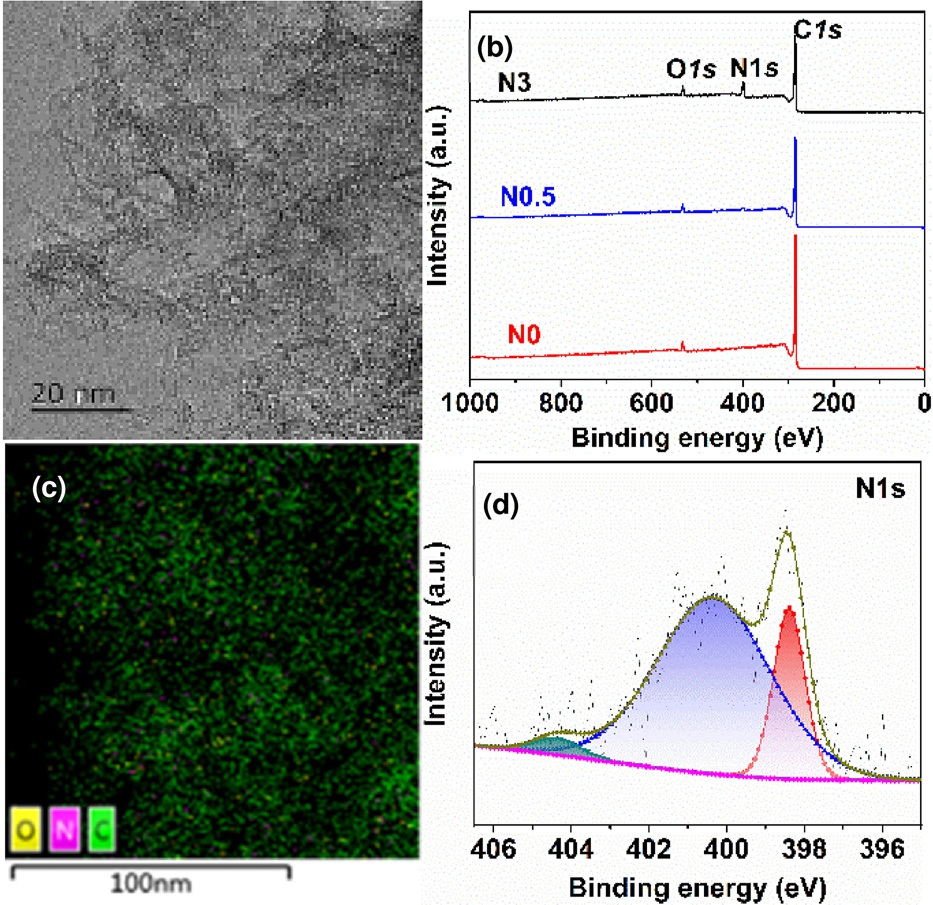
a) TEM image and c) elemental mapping of N0.5. b) XPS survey scan and d) high‐resolution N 1s spectra of N0.5.

Inspired by the reported high activity in the acetylene hydrochlorination and EDC dehydrochlorination reactions,^[6]^ N‐doped carbon was chosen for testing the VCM production. The N‐doped mesopores carbon was synthesized by polymerization with melamine (M) as the nitrogen precursor, phenol (P), and formaldehyde (F) as the carbon source, using SiO_2_ colloid as a hard template following the carbonizations and removal of the templates. To achieve different N contents, different precursor compositions (Nx, *x* stands for the mol ratio of M/P) were used. The morphologies of the N‐doped mesopores carbon were characterized by the high‐resolution transmission electron microscopy (TEM), as shown in Figure 1, Brunauer–Emmett–Teller surface area (Supporting Information, Figure S1 and Table S1), and X‐ray diffraction (XRD; Figure S2) measurements. The very broad diffraction peaks on the XRD patterns indicated the amorphous structure and no obvious structural difference with the different N contents.^[7]^ Raman spectra show typical characteristic D and G bands of disordered graphitic materials (Figure S3), the intensity ratios of *I*_D_/*I*_G_ increased with increasing the N content, indicating the increased defectiveness. Thus, from XRD and Raman, it can be concluded that doping N can introduce disorder in the graphitic structure. The mesopores with the diameters range of 10–50 nm are observed on the N0.5 catalyst. From the TEM‐mappings of the carbon catalysts (Figures S4–S6), N was introduced into the mesopores carbon, and it is highly dispersed in the carbon, which contributed to the high specific surface area and pore volume.^[8]^ The introduction of N into the carbon was also confirmed by the X‐ray photoelectron spectroscopy (XPS) analysis. A high‐resolution XPS scan was applied to shed light on the N species in the mesopores carbon (Figure 1 d; Figure S7). The deconvolution of the N 1s spectra of N0.5 indicated that three N species existed in the mesopores carbon corresponding to pyridinic N, pyrrolic N, graphitic N^[8]^ (Tables S2 and S3), where the pyridinic N and pyrrolic N are most prevalent.

(1) $$C{}_{2}H{}_{4}+0.5O{}_{2}+2\mathrm{HCl}\to C{}_{2}H{}_{4}\mathrm{Cl}{}_{2}+H{}_{2}O;\;\Delta H=-239.7\;\mathrm{kJ}\;\mathrm{mol}{}^{-1}$$

(2) $$C{}_{2}H{}_{4}\mathrm{Cl}{}_{2}\to C{}_{2}H{}_{3}\mathrm{Cl}+\mathrm{HCl};\;\Delta H=73.0\;\mathrm{kJ}\;\mathrm{mol}{}^{-1}$$

(3) $$C{}_{2}H{}_{4}+0.5O{}_{2}+\mathrm{HCl}\to C{}_{2}H{}_{3}\mathrm{Cl}+H{}_{2}O;\;\Delta H=-166.7\;\mathrm{kJ}\;\mathrm{mol}{}^{-1}$$

Ethylene oxychlorination, EDC dehydrochlorination, and VCM direct formation from ethylene oxychlorination are presented in Equations (1)–(3). Thermodynamic calculations for the Equations (1)–(3) were performed (Figure S8). EDC dehydrochlorination reaction [Eq. (2)] is highly endothermic and Gibbs free energy is negative only at higher temperatures (>≈250 °C). The Δ_r_ *G* is highly negative for reaction Equation (3) leading to direct VCM formation, which is combined reactions of 1 and 2, suggesting the reaction highly thermodynamically favorable.

**Figure 2:**
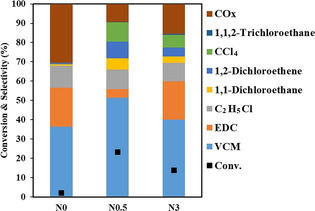
Catalytic results of the N‐doped carbon catalysts for ethylene oxychlorination. Reaction conditions: C_2_H_4_/O_2_/HCl=2:1:2, 250 °C, *W*_cat_ (0.8 g), gas hour space velocity: 4350 mL h^−1^ g_cat_, *F* ${}_{C{}_{2}H{}_{4}}$ =4 mL min^−1^, *P*_total_=1 bar.

The catalyst testing was performed in a fixed‐bed reactor at 250 °C using the stoichiometric C_2_H_4_: O_2_: HCl ratio of 2:1:2 following the reaction [Eq. (3)] on carbon catalysts (N0, N0.5 and N3). The conversion and product distribution are presented in Figure 2, compared to the carbon catalyst without N doping (N0). The carbon catalyst (N0) is almost inactive, while metal‐free N‐doped carbon is highly active for ethylene oxychlorination direct to EDC and VCM. Based on our best knowledge, this is the first time to observe the metal‐free N‐doped carbon catalyst active for C_2_H_4_ oxychlorination and make it possible for a one‐pot synthesis of VCM at low temperatures. The metal‐free catalyst has a remarkably higher low‐temperature activity compared to Lanthanide compounds which are typically active only at temperatures higher than 400 °C.^[5]^ The metal‐free catalyst is highly stable and no changes in the activity and selectivity were observed during the 40 h time on stream (Figure S9). The main products are VCM, EDC, byproducts of CO_x_ (CO and CO_2_), and other chlorinated products, where VCM is dominating. The selectivity depends on the N content and selectivity to VCM (≈50 %) on N0.5 is highest among the catalysts tested.

Given the unprecedented activity of N‐doped carbon for both oxychlorination and dehydrochlorination, kinetic study, and various temperature‐programmed desorption (TPD) and temperature‐programmed surface reactions (TPSR) were performed to elucidate the reaction network, reaction mechanism, and active sites.

**Figure 3:**
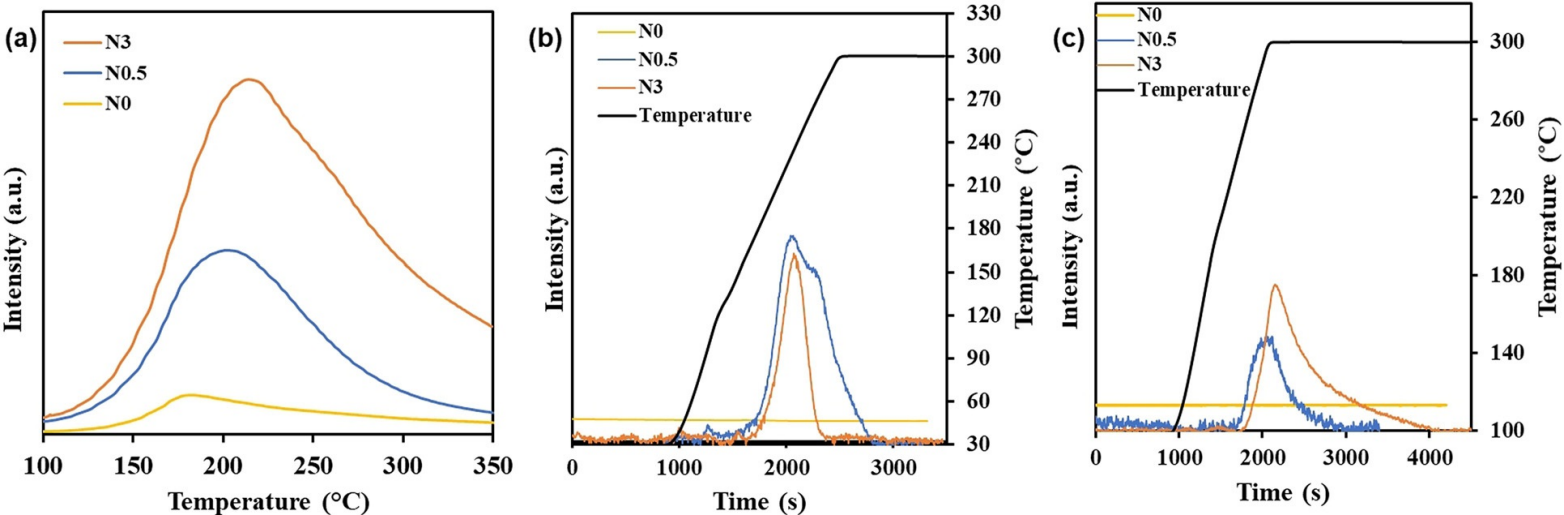
TP‐X profiles for the N‐doped catalysts, *P*_total_=1 bar, *W*_cat_=0.5 g, 10 °C min^−1^. a) HCl‐TPD, *F*_Ar_=100 mL min^−1^. b) C_2_H_4_/O_2_‐TPSR after saturated adsorbing of HCl. The signal of VCM was recorded under the following conditions: *F*_total_=100 mL min^−1^, *F* ${}_{C{}_{2}H{}_{4}}$ =10 mL min^−1^, C_2_H_4_/O_2_=2, diluted in Ar. c) EDC‐TPSR with HCl recorded by MS, *F*_Ar_=100 mL min^−1^.

The HCl‐TPD (Figure 3 a) illustrated the adsorbed amount of HCl increases with increasing the N content. Without N doping, only a very weak desorption peak at 175 °C was observed. It rationalizes that almost no activity was observed on the neat carbon surface. When it is doped with N, not only the desorbed amount of HCl increased but also the desorption temperature shifts to higher ones with increasing the N content, suggesting stronger adsorption of HCl on catalysts with the higher content of N. Density function theory (DFT) calculations suggested chemisorption of HCl on N groups forming N−H−Cl.^[9]^ The acidic strength follows an order of pyridinic >pyrrolic >graphitic N groups, and the graphitic N does almost not contribute to the basicity of the catalyst.^[10]^ The higher N content, the more HCl was desorbed with higher desorption temperature, possibly attributed to the higher number of the pyridinic N sites (Table S4) with stronger basicity.^[10a]^ C_2_H_4_‐TPD (Figure S10) suggests the ethylene can adsorb rather strongly on the carbon surface. The literature suggests also that oxygen can be strongly adsorbed on the carbon surface.^[11]^

The active site requirement is further examined by analyzing the contributions of different N groups to the catalytic performance. Although, several methods have been reported for synthesis N‐doped carbon catalysts,^[12]^ multiple types of N species were often introduced simultaneously. It makes the identification of active sites in N‐doped carbon very challenging. However, the fact that the higher N content (N3) yielded a lower reaction rate than N0.5 (Figures 2 and 3 b) suggests that the ethylene oxychlorination is not directly related to the total N content. As we discussed above, three N groups exist and the distribution of the N groups varies with the catalysts (Figure 1 d; Figure S7, Tables S3–5). We normalized the reaction rate of ethylene oxychlorination based on the number of three N species, as shown in Table S5. Only the normalized ethylene reaction rates based on the pyrrolic N group are similar, and others have almost two orders of magnitude difference. It suggests that the pyrrolic N might be a highly relevant active site for the ethylene oxychlorination, which is similar to the previous reports[^6a^, ^6d^] that the pyrrolic N sites on N‐doped carbon catalysts contribute mainly to the acetylene hydrochlorination.

The exact active site for catalytic EDC dehydrochlorination on N‐doped carbon is still in debate, where pyridinic and pyrrolic N or pyridinic and graphitic N groups as main active sites have been reported.^[13]^ The activity of EDC dehydrochlorination depends on the basicity. Combining the facts of more EDC adsorbed, more VCM formed, and the lower starting temperature of the TPSR of EDC on N3 compared to N0.5 and N0, the activity of EDC dehydrochlorination follows the order of the N content. When looking at the number of N species (Table S4), both pyridinic and graphitic N contents increased with increasing N content. Owing to the very weak basicity of graphitic N, it can be proposed that pyridinic N groups with the highest basicity among the N groups could be the main active sites for EDC dehydrochlorination.

The selectivity vs. conversion plot (Figure S11) suggests EDC is an unstable primary product, while VCM is the primary product plus the secondary product from EDC dehydrochlorination. The possible contribution of Cl_2_ from the Deacon reaction (4 HCl + O_2_ = 2 Cl_2_ + 2 H_2_O) to EDC formation was excluded experimentally (Section S5 in supporting information).

**Figure 4:**
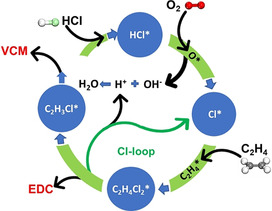
The reaction cycles that produce VCM in a single step from ethylene oxychlorination on the N‐doped carbon catalysts.

The reactivity of adsorbed HCl was checked by the TPSR of C_2_H_4_/O_2_ on HCl saturated surfaces (Figure 3 b; Figures S12 and S13). VCM was produced dominatingly on the N‐doped carbon catalyst in addition to HCl desorption and ethyl chloride (EC) production (Figures S12 and S13); while no VCM was detected on the N0 catalyst. Moreover, TPSR of ethylene in the absence of oxygen on the HCl saturated adsorbed surfaces produced only EC (Figure S14). EC is also an unstable primary product in ethylene oxychlorination (Figure S11). However, the possible contribution in VCM formation by the oxidative dehydrogenation of EC has been excluded by the reaction of EC with O_2_, which formed mainly ethylene instead of VCM (Figure S15). A comparison between TPSR of C_2_H_4_/O_2_ and TPSR of C_2_H_4_ reveals that oxygen is essential to provide the surface Cl* for chlorinating ethylene to EDC, which is most likely obtained from the oxidative dissociation of the adsorbed HCl*. The VCM is also a product of the catalytic dehydrochlorination of EDC, which was evidenced by TPSR of EDC where the adsorbed EDC was selectively converted to VCM, (Figure 3 c). By summarizing all the results, a reaction mechanism is proposed, and a detailed discussion of the reaction mechanism can be seen in the supporting information (Section 5). The catalytic cycle leading to EDC and VCM on the N‐doped carbon surface is presented in Figure 4.

The metal‐free N‐doped carbon catalyst has the redox activity for ethylene oxychlorination as well as the activity for the dehydrochlorination. The catalyst surface has bi‐functionality and both reactions can occur on the same type of active site. The reaction follows a Langmuir‐Hinshelwood mechanism. All the reactants, C_2_H_4_, HCl, and O_2_ adsorbed on the surface. The adsorbed HCl oxidatively dissociates to form the surface Cl*. The surface Cl* chlorinated the adsorbed ethylene to form adsorbed EDC, which can be desorbed to gas‐phase EDC or further go to dehydrochlorination. The EDC dehydrochlorination involves C−H and C−Cl cleavage to form surface H* and Cl*.^[14]^ Results of VCM being the primary product of oxychlorination, full conversion to VCM in TPSR of C_2_H_4_/O_2_, and VCM formed in TPSR of EDC reveal a Cl* loop between the ethylene oxychlorination and EDC dehydrochlorination, instead of a gas phase HCl loop. The surface Cl* generated by EDC* dehydrochlorination directly participates in the ethylene oxychlorination. Therefore, both EDC and VCM are primary products.

However, the N‐doped carbon catalyst has a drawback with relatively high selectivity of CO_*x*_. The possible contribution of the oxidation of the N‐doped carbon catalyst itself in the formation of CO_*x*_ at the reaction conditions was eliminated by the temperature‐programmed oxidation of the carbon catalysts, no CO_2_ was detected until 400 °C (Figure S16). It indicated that the catalyst materials can be stable under the reaction condition (250 °C). It is well known that certain surface oxygen groups on the carbon surface can catalyze the deep oxidation to lead CO_*x*_ formation.[^11^, ^15^] The challenge on selectivity to CO_*x*_ on carbon‐based catalysts has long been documented in a similar type of redox reaction such as oxidative dehydrogenation of hydrocarbons. The oxygen functional groups on N0.5 were characterized by the high‐resolution O 1s XPS scan (Figure S17). The peaks were assigned to C‐O with the binding energies of ≈532.6 eV and C=O with the binding energy of ≈531.3 eV. The C=O surface oxygen groups have been recognized as the main group contributing to the overoxidation of the reactants and products in the presence of oxygen molecules to form the CO_*x*_.^[15c]^ It should be noted that N‐doped carbon has not been optimized further and the material could be modified by P or B to reduce the formation of CO_*x*_.^[11]^

**Figure 5:**
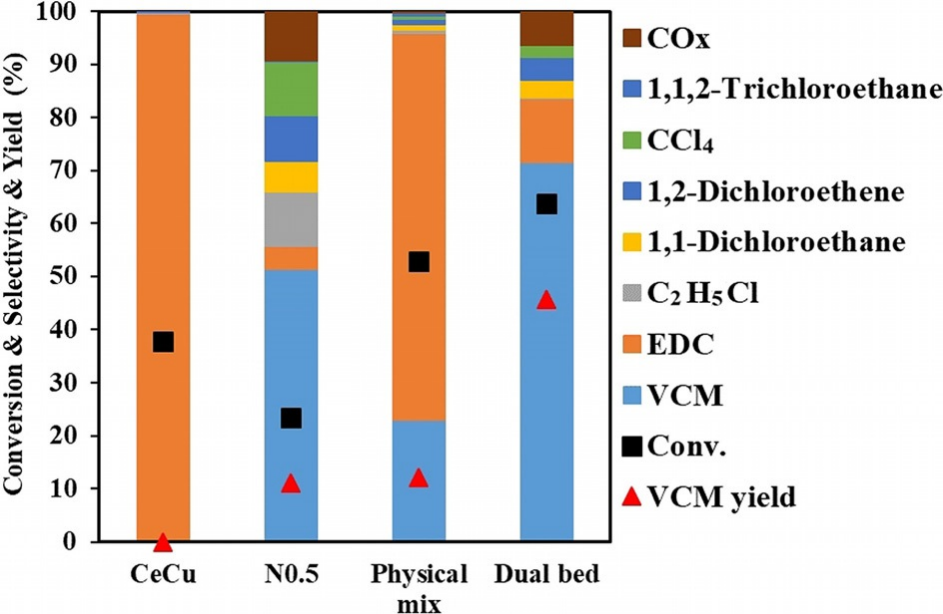
Catalytic results in comparison (CeCu/Al_2_O_3_ at the top, N0.5 at the bottom) at the same flow rate. Reaction conditions: C_2_H_4_/O_2_/HCl=2:1:2, 250 °C, CeCu (0.2 g), N0.5 (0.8 g), for physical and dual bed catalysts mass CeCu (0.2 g) + N0.5 (0.8 g), *F* ${}_{C{}_{2}H{}_{4}}$ =4 mL min^−1^, *P*_total_=1 bar.

Based on the above discussion, lowering the oxygen concentration in the gas phase exposing to the carbon surface could potentially reduce the CO_*x*_ formation. A tandem reaction on a bi‐functional catalyst strategy was then proposed and tested, where the CuCl_2_/Al_2_O_3_‐based oxychlorination catalyst and the N‐doped carbon are installed together. The promoted CuCl_2_/Al_2_O_3_ catalysts are typical industrial catalysts for EDC production.[^1a^, ^4^] The Ce‐promoted CuCl_2_/Al_2_O_3_ catalyst has been reported to be active and stable for the ethylene oxychlorination.^[4e]^ Figure 5 shows very high selectivity to EDC of above 99 %, and no VCM on the Ce‐promoted CuCl_2_/Al_2_O_3_ catalyst, which is consistent with the previous report.^[4e]^ The activity of CuCl_2_/Al_2_O_3_ catalysts is higher compared to the N‐doped carbon catalyst, N0.5 (Figure 5). In addition to high activity for ethylene oxychlorination, N‐doped carbon showed also high activity, selectivity, and stability for EDC dehydrochlorination (Figure S18), in good agreement with literature observations.[^6b^, ^13^] No deactivation was observed during the 12 h at 250 °C for EDC dehydrochlorination on the N0.5 catalyst.

Two bifunctional catalyst configurations, namely a physical mixture of two catalysts and dual‐bed with CuCl_2_/Al_2_O_3_ catalysts at the top following the N0.5 catalyst, are tested for the tandem reactions. As shown in Figure 5, the tandem reactions on the physically mixed catalysts reduced significantly the CO_*x*_ selectivity. However, only 23 % selectivity of VCM was obtained, and the EDC is still the main product. Moreover, the conversion on the mixed bed lowered about 8 % of the sum of the conversion from the individual CuCl_2_/Al_2_O_3_ and N‐doped carbon beds. It can be ascribed by the strong adsorption of the chloride‐containing compounds such as VCM on the CuCl_2_‐based catalyst surface, suppressing the oxychlorination reaction, as shown in Figure S19. In the dual‐bed configuration, the high selectivity of VCM of about 70 % and low selectivity to CO_*x*_ (≈6 %) were obtained. Part of the O_2_ was consumed in the oxychlorination reaction on CeCu/Al_2_O_3_, and oxygen concentration is relatively low in the second catalyst layer, confirmed the above hypothesis of decreasing the CO_*x*_ formation by reducing the oxygen concentration exposed to the carbon surface. A VCM yield of 45.6 % at a C_2_H_4_ conversion of 63.7 % was obtained at 250 °C and 1 bar, which is comparable to the current industrial single‐pass two‐step process as discussed above. Furthermore, the C_2_H_4_ conversion on the dual‐bed catalysts is much higher than that on the physical mixture of the catalysts, and even slightly higher than the sum of the conversion on the neat CeCu/Al_2_O_3_ and the N‐doped carbon catalysts. Moreover, the VCM selectivity is much higher in the dual bed compared to the physical mixture. The enhanced ethylene conversion in the dual‐bed is ascribed to the synergy of the EDC dehydrochlorination and ethylene oxychlorination on the bi‐functional N‐doped carbon surface. In the first catalyst bed, the most of HCl was consumed by ethylene oxychlorination, while in the second catalyst bed both the EDC dehydrochlorination and ethylene oxychlorination were coupled together on the N‐doped carbon surface through the surface Cl* loop, generating a synergy effect between the two reactions. The effects of the N species of the N‐doped carbon in the second layer on the performance of the dual‐bed catalysts are presented in Figures S20 and 21. VCM/EDC ratio follows an order of N0.5 > N3 > N0, in good agreement with the activity order of ethylene oxychlorination on the same type of catalysts. The multifunctionality, relatively high number of pyrrolic and pyridinic species of N0.5 makes it possible to effectively combine both the ethylene oxychlorination and EDC dehydrochlorination reactions on the surface to lead a high yield of VCM.

**Figure 6:**
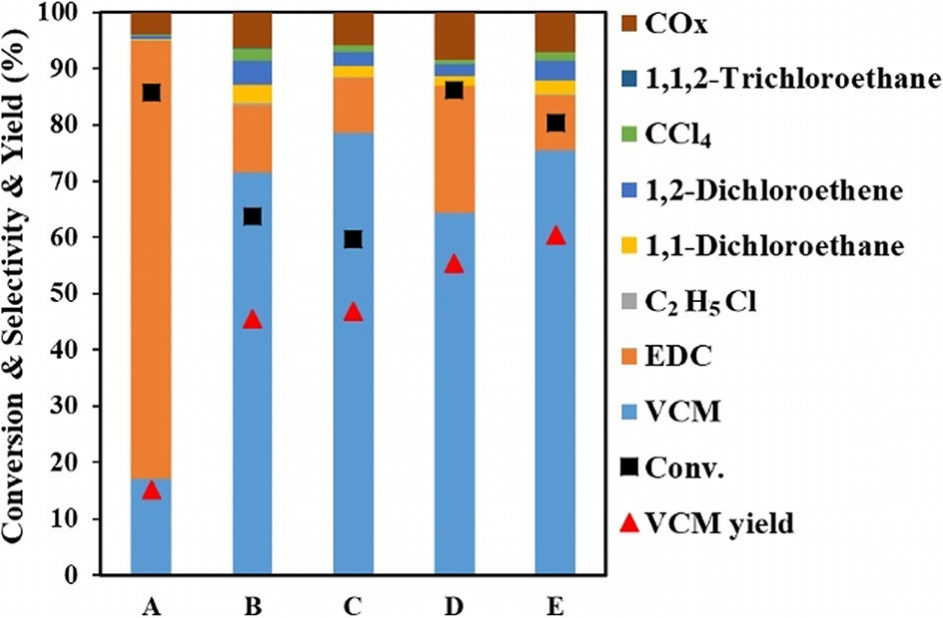
Catalytic results of the dual‐bed method (CeCu/Al_2_O_3_ at the top, N0.5 at the bottom) with different mass ratios at the same conditions: A) 1:1, B) 1:4, C) 1:9, D) 1:2, E) 1:5; with a total mass of 1 g (A–C) and 1.2 g (D,E). Reaction conditions: C_2_H_4_/O_2_/HCl=2:1:2, 250 °C, *F* ${}_{C{}_{2}H{}_{4}}$ =4 mL min^−1^, *P*_total_=1 bar.

Moreover, the bifunctionality of the N‐doped carbon leads to the direct production of VCM from the ethylene oxychlorination. In principle, it breaks down the constrain of the chemical equilibrium of the EDC dehydrochlorination at low temperatures. Therefore, the tandem reaction using bi‐functional N‐doped carbon can intensify reactions toward VCM formation at relatively low temperatures. The tandem reaction was then optimized to enhance the yield of VCM by tuning the mass ratio of the two catalysts in the dual‐bed (Figure 6). Conditions A, B, and C are varying the ratio with the total mass kept. The results clearly showed that more N‐doped carbon catalyst in the reactor, the higher the VCM selectivity is. The higher VCM yield of 60 % due to a higher conversion of ethylene and selectivity to VCM at the condition E was obtained. Further increase the residence time the VCM yield increased up to 75.7 % (Table S6), with the C_2_H_4_ conversion of 94.8 %. The results revealed that the VCM yield depends on the residence time and the ratio of the catalysts. The VCM yield is expected to be further optimized through the modification of N‐doped carbon catalysts.

## Conclusion

Herein we have reported a metal‐free N‐doped carbon as an efficient bifunctional catalyst of ethylene oxychlorination and EDC dehydrochlorination for the synthesis of VCM and EDC at low temperatures (around 250 °C). The pyrrolic and pyridinic N were proved to be the main active sites on the N‐doped carbon for ethylene oxychlorination and EDC dehydrochlorination, respectively. A reaction mechanism with surface Cl* looping between ethylene oxychlorination and EDC dehydrochlorination was proposed to rationalize the observed synergy effect of the two reactions. Moreover, we demonstrated an efficient one‐pot dual‐bed bifunctional catalyst concept to produce VCM with a yield up to 76 % under mild conditions of 250 °C, 1 bar, and a stoichiometric C_2_H_4_:O_2_:HCl ratio of 2:1:2, much higher (ca. 25 %) than that in the current two‐step industrial process in a single‐pass. In the first bed, CuCl_2_/Al_2_O_3_‐based catalyst catalyzes the ethylene oxychlorination, while in the second bed N‐doped carbon coupled both ethylene oxychlorination and EDC dehydrochlorination reactions together on the surface. We believe that the metal‐free N‐doped carbon catalysts could have a significant impact on VCM‐related studies for both academia and industry toward a more cost‐ and energy‐effective VCM production process.

## Conflict of interest

The authors declare no conflict of interest.

## Supporting information

As a service to our authors and readers, this journal provides supporting information supplied by the authors. Such materials are peer reviewed and may be re‐organized for online delivery, but are not copy‐edited or typeset. Technical support issues arising from supporting information (other than missing files) should be addressed to the authors.

Supplementary
