# Supplementary material for: Nitrogen‐Doped Carbon‐Assisted One‐pot Tandem Reaction for Vinyl Chloride Production via Ethylene Oxychlorination
Source: Angew Chem Int Ed Engl. 2020 Sep 29;59(49):22080–5. doi: 10.1002/anie.202006729 (PMC7756741; doi:10.1002/anie.202006729)
Supplement: Supplementary file 1 — Supplementary [file ANIE-59-22080-s001.pdf]

Angewandte Chemie
Eine Zeitschrift der Gesellschaft Deutscher Chemiker

Supporting Information

Nitrogen-Doped Carbon-Assisted One-pot Tandem Reaction for Vinyl Chloride Production via Ethylene Oxychlorination

*Hongfei Ma, Guoyan Ma, Yanying Qi, Yalan Wang, Qingjun Chen, Kumar R. Rout, Terje Fuglerud, and De Chen**

anie_202006729_sm_miscellaneous_information.pdf

Author Contributions

H.M. Conceptualization: Lead; Formal analysis: Lead; Writing—Original Draft: Lead; Writing—Review & Editing: Lead
G.M. Conceptualization: Supporting; Funding acquisition: Supporting
D.C. Formal analysis: Lead; Funding acquisition: Lead; Investigation: Lead; Supervision: Lead; Writing—Review & Editing: Lead.

Table of Contents

S1. Experimental Procedures

Catalyst preparation

Catalyst characterization

Catalytic performance evaluation

S2. Catalyst characterization and properties

Figure S1. N_2 adsorption-desorption isotherms of the carbon catalysts.
Figure S2. XRD patterns of the carbon catalysts.
Figure S3. Raman spectra of the carbon catalysts.
Figure S4. HR-TEM images with the corresponding elemental mapping of the N0 catalyst.
Figure S5. HR-TEM images with the corresponding elemental mapping of the N0.5 catalyst.
Figure S6. HR-TEM images with the corresponding elemental mapping of the N3 catalyst.
Figure S7. N1s XPS spectra of N3.

Table S1. Pore parameters of the N-doped mesopores carbon.
Table S2. Elements concentration in the N-doped carbon catalysts.
Table S3. Relative N species distribution in the N-doped carbon catalysts.
Table S4. The absolute amount of N species distribution in the N-doped carbon catalysts (g/g_{cat}).

S3: Thermodynamic analysis

Figure S8. The Gibbs Energy ($\Delta_r G$) as a function of temperature for the related reactions, calculated by HSC 6.0 software.

S4: Catalytic performance

Figure S9. Catalytic results of the N0.5 catalyst for ethylene oxychlorination.

Figure S10. C_2H_4-TPD profiles on the carbon catalysts.

Figure S11. Product selectivity vs. ethylene conversion at 250 °C and 1 bar with the feeding molar ratio of 2:1:2 on N0.5.

Figure S12. C_2H_4/O_2 TPSR of N0.5 after saturated adsorbing of HCl.

Figure S13. C_2H_4/O_2 TPSR of N3 after saturated adsorbing of HCl.

Figure S14. C_2H_4 TPSR of N0.5 after saturated adsorbing of HCl.

Figure S15. GC FID result of the ethyl chloride oxidation on N0.5.

Figure S16. CO_2 formation of the carbon catalysts in O_2.

Figure S17. O1s XPS spectra of all the carbon catalysts.

Figure S18. a) EDC conversion versus temperature in the dehydrochlorination of EDC by the bubbling method using He as the carrier gas. b) Stability test at 250 °C on N0.5 catalyst.

Figure S19. Catalytic results of the ethylene oxychlorination on the CeCu/Al_2O_3 catalyst.

Figure S20. a) Catalytic results of the dual-bed catalyst (CeCu/Al_2O_3 at the top, N-doped carbon at the bottom), b) VCM-to-EDC ratio.

Figure S21. a) Catalytic results of the dual-bed catalyst (CeCu/Al_2O_3 at the top, N-doped carbon at the bottom), b) VCM-to-EDC ratio.

Table S5. The ethylene reaction rate of ethylene oxychlorination calculated based on the N-species (pyridinic, pyrrolic, and graphitic).

Table S6. Catalytic results of the dual-bed method (0.5 g CeCu/Al_2O_3 at the top, 1.5 g N0.5 at the bottom).

S5: Reaction network and reaction mechanism

S1. Experimental Procedures

Catalyst preparation.

The $CuCl_2/\gamma$-Al_2O_3-based oxychlorination catalyst was prepared by the incipient wetness method as described in our previous report.[1] The precursors $CuCl_2$ and $CeCl_3$ solution were impregnated on the Al_2O_3 with the Cu loading of 5 wt%, and the molar ratio of 0.4 for Ce/Cu. After impregnation, the samples were put in the oven at room temperature for 10 h, followed by heating to 120 °C with the ramping rate of 2 °C/min and kept for 6 h. The obtained samples were sieved to 45−100 μm before use.

Nitrogen-doped carbon catalysts were synthesized by a hard template method, in which formaldehyde (F, 37 wt%) and phenol (P) were used as carbon precursors, melamine (M) as the nitrogen source, and SiO_2 nanoparticles as the hard template. Firstly, F and P were added together into 100 ml 0.2 M NaOH solution with a molar ratio of 2. The mixture was stirred at 700 °C for 40 min, followed by adding F and M into the solution and stirred for another 30 min. Then Ludox SM-30 sol (30% SiO_2) was added into the mixture during stirring. The resulting solution was placed in an oil bath at 85 °C for 5 days to form an organic gel. The gel was then dried at room temperature for 2 days at 120 °C for 24 h. The obtained gel was then carbonized at 700 °C for 4 h in the Ar atmosphere at a ramping rate of 5 °C/min. The carbon/SiO_2 composite was submerged in 3M KOH at 85 °C overnight to remove the silica. Finally, the obtaining samples were washed until the pH was 7 and then dried at 100 °C to dry. The last sample obtained was nitrogen-doped carbon. The molar ratio of M/P was tuned to get different N contents. Higher M/P ratio of higher nitrogen content. The resulting samples were named as Nx, where x is the molar ratio of M/P.

Catalyst characterization

The specific surface area, pore volume and pore size of the catalysts were measured on a TriStar 3020 instrument using N_2 isotherms adsorption at 77 K, calculated with the respective BET and BJH methods. The samples were degassed at 120 °C overnight before the BET measurements.

XRD profiles were recorded on a Bruker D8 Advanced DaVinci X-ray diffractometer using Cu Kα1 and 0.15 nm wavelength.

The high-resolution of transmission electron microscopy (HR-TEM) was performed on a JEM-2100F Microscope operated at an accelerating voltage of 200 kV. The sample powders were well dispersed in ethanol under ultrasonic treatment before the measurement. Energy dispersive X-ray (EDX) analysis of the catalysts was performed using the same instrument.
The surface analysis and N (and O) species were performed by X-ray photoelectron spectroscopy (XPS) using a ThermoFisher K-alpha X-ray photoelectron spectrometer system, equipped with monochromator Al Kα radiation. The C1s peak at 284.8 eV was used as a calibration for the other peaks.
The Raman spectroscopy was performed on LabRAM HR800, using a Ne-Ne laser in the visible range (633 nm), with 1800 gr/mm and a hole of 200 μm.

Catalytic performance evaluation

The catalytic performance evaluation was performed in a fixed-bed glass reactor. The catalyst was heated to the target reaction temperature with a rate of 10 °C/min in Ar. The reactant gases (C_2H_4: pure, AGA, 3.5, 20% O_2 in Ar: AGA 5.0, 20% HCl in Ar: AGA 5.0, N_2: pure, AGA 5.0) were fed into the reactor with calibrated mass flow controllers. All the reactions were performed at atmospheric pressure. The products were analyzed by an on-line Agilent 7890B GC, which was equipped with a thermal conductivity detector (TCD) and a flame ionization detector. Chlorine-containing compounds were analyzed by FID, while CO_2, CO, C_2H_4, and N_2 were analyzed by TCD. Herein, N_2 was used as an internal standard for analysis. Generally, the reaction was conducted under the condition of 250 °C and ambient pressure, with the ethylene flow rate of 4 ml/min and a total flow rate of 58 ml/min.
The C_2H_4 conversion and product selectivity were calculated with the following equations, where C_i denotes the molar fraction of a product *i*. the vi means the stoichiometric parameter for the converting of C2H4 to the product *i* to the number of carbon atom numbers, for example, $\mathrm{C_2H_4 + 3O_2 \rightarrow 2CO_2 + 2H_2O}$, v_i=2.

$$X_{C_2H_4} = \frac{C_2H_{4_{intlet}} - C_2H_{4_{ontlet}}}{C_2H_{4_{intlet}}} \times 100\%$$

$$S_i = \frac{C_i}{\sum C_i/v_i} \times 100\%$$

For the ethylene oxychlorination reaction on the $CuCl_2/Al_2O_3$-based catalyst (0.2 g), before co-feeding the reactant gas, the catalyst was treated in HCl gas to get rid of the paratacamite (due to be stored in the air).

For the physical mixture of the catalysts, the $CuCl_2/Al_2O_3$-based catalyst and N-doped carbon were physically mixed with the specific mass ratios (0.2 g and 0.8 g) before mounting into the reactor.
As for the dual bed reactor, the N-doped carbon catalyst was put at the bottom of the reactor, the $CuCl_2/Al_2O_3$-based catalyst was put on the top layer of the reactor, the two layers were separated by the glass wool.
In the conversion vs. selectivity test on the N0.5 (1 g) catalyst, the space velocity was altered, while keeping the molar ratio of $C_2H_4/O_2/HCl=2:1:2$.
EDC cracking was also performed on the same setup with the fix-bed reactor. The catalyst (0.5 g) was first heated to the target reaction temperature, followed by introducing EDC (Sigma Aldrich, 99.8%) through bubbling by He as the carrier gas.
In the Deacon reaction HCl and O_2 were introduced into the N-doped carbon (N0.5, 0.5 g) catalyst with a molar ratio of 4:1. The gas stream flowed to an adsorption bottle containing an aqueous KI solution (Sigma Aldrich, 0.1 mol/L, 100 ml) for iodometric titration. This test was used to verify the production of Cl_2.
In the ethyl chloride oxidation reaction on the N-doped carbon catalyst, C_2H_5Cl (AGA, 1% in Ar) and O_2 were introduced into the catalyst at 250 °C and 1 bar. Herein, it is noted that 1% C_2H_5Cl is the maximum limit that the gas supplier (AGA) can provide. The catalyst was heated in Ar to 250 °C with a ramping rate of 10 °C/min. Then, the C_2H_5Cl and O_2 were introduced into the reactor, and the product was analyzed on the online GC, which is equipped with TCD and FID.

HCl temperature-programmed desorption (HCl-TPD)

HCl-TPD was performed on our home-made setups combined with an MS to record the signal of HCl. The samples (0.5 g) were firstly treated at 100 °C in Ar to purge out the moisture for 1 h and cool down to 30 °C. The samples were then saturated with 20% HCl/Ar for 1 h. Subsequently, the samples were purged with Ar in 100 °C for 1h to remove out the physical adsorbed HCl with the flow rate of 30 ml/min. Once a stable baseline was obtained, chemisorbed HCl was desorbed by heating from 100 °C to 350 °C with a ramping rate of 10 °C/min with the effluent gas monitored by an online MS (Hiden Analytical HPR-20 R&D). The final temperature was kept for 30 min for a stable signal.

C_2H_4 temperature-programmed desorption (C_2H_4-TPD)

C_2H_4-TPD was performed similarly with the step discussed above. The samples (0.5 g) were firstly treated at 100 °C in Ar to purge out the moisture and cool down to 30 °C. The samples were then saturated with 20% C_2H_4/Ar for 1 h. Subsequently, the samples were purged with Ar in 100 °C to remove the physical adsorbed C_2H_4 with a flow rate of 50 ml/min. Once a stable baseline was obtained, chemisorbed C_2H_4 was desorbed by heating from 100 °C to 350 °C with a rate of 10 °C/min with the effluent gas monitored by an online MS (Hiden Analytical HPR-20 R&D). The final temperature was kept for 30 min until a stable signal was obtained.

Temperature programmed surface reaction (TPSR)

All the TPSR experiments were also performed on the same setup with an online MS (Hiden Analytical HPR-20 R&D) recording the gas effluent. The samples (1 g) were heated in Ar to 100 °C and stayed for 1 h to purge out the adsorbed moisture. Then, the samples were cooling down to room temperature, diluted HCl (20% in Ar) gas was flowed through the catalysts and adsorbing at least 1 h. The samples were purged by Ar to purge out the physically adsorbed HCl. When a stable MS baseline was obtained, C_2H_4 and O_2 were switched to the catalyst until a stable MS signal was observed. Then TPSR was performed by heating the samples in C_2H_4 and O_2 with Ar at a ramping rate of 10 °C/min with the total flow rate of 100 ml/min, and C_2H_4/O_2 ratio of 2:1. The effluent gas was recorded by an online MS.

A similar procedure was performed for the C_2H_4-TPSR, except that, only 10% C_2H_4 (no O_2) was introduced into the catalyst during the heating process.

The temperature-programmed desorption of EDC (EDC-TPD) was also performed on the same setup with an online MS. Since EDC would be dehydrochlorinated to VCM during heating, it can also be called TPSR. The samples (0.5 g) were pretreated at 100 °C in the Ar atmosphere to purge out the adsorbed moisture. EDC was introduced by bubbling using Ar as the carrier gas at room temperature for 1 h. Then the samples were heated to 100 °C in Ar to purge out the physical adsorbed EDC and stayed for 1 h. Then TPD was performed by heating the catalyst to 300 °C at a ramping rate of 10 °C/min in Ar with the flow rate of 100 ml/min. During heating, EDC would be dehydrochlorinated to VCM and HCl, so the signal of HCl was recorded by an online MS for analysis.

TGA

TGA was performed to evaluate the stability of the catalyst on Linseis STA PT1600. The catalysts (0.05 g) were treated in Ar for 30 min at 30 °C to remove the adsorbed moisture. Then the catalysts were treated in 6% O_2 in Ar to 800 °C with the ramping rate of 5 °C/min, with an online MS recording the CO_2 signal. The temperature was kept for at least 5 min until a stable baseline was obtained.

S2. Catalyst characterization and properties

The pore structures of the N-doped carbon were measured by N_2 physisorption. The BET surface area, BJH pore size distribution, and pore parameters are summarized in Table 1. The samples exhibited type-IV isotherms, combined with the pore size, revealing the typical mesoporous structures.[2] Doping N into the carbon, surface area, pore volume, and pore size increased a little bit. These findings suggested that melamine has a positive effect on the pore structure of the mesoporous carbon. While, the samples after the catalytic reaction were also tested, as shown in the column of spent in Table 1. The surface area and pore volume were slightly decreased compared with the fresh samples. But the pore size was slightly increased in all the samples.

The N concentration was identified by EDX with multiarea for better verification, while the type and fraction of different N species were determined by XPS. It is commonly used in the N-doped materials in a wide range of literature (actually, most of the researches is using the XPS technique). In the XPS tests, each component was tested three times, the spectra were averaged for the analysis.

Table S1**.** Pore parameters of the N-doped mesopores carbon.

Catalyst	Surface area (m^2/g)		Pore volume (cm^3/g)		Pore size (Å)	
	Fresh	Spent	Fresh	Spent	Fresh	Spent
N0	822	667	1.7	1.5	96.3	109.6
N0.5	886	731	2.3	2.1	99.3	113.5
N3	1058	913	3.2	3.0	108.6	129.3

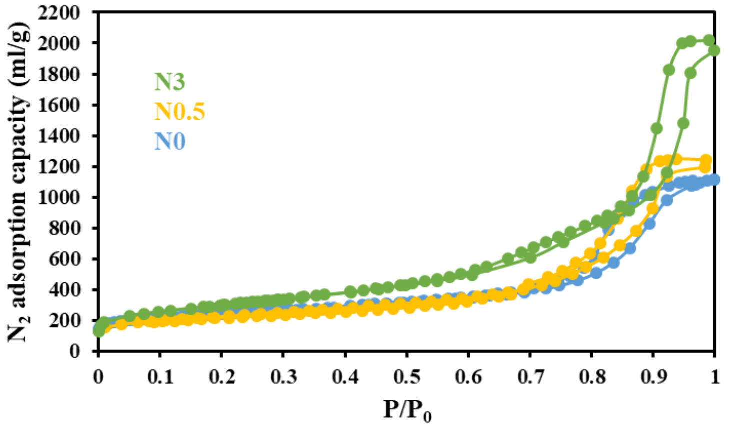

Figure S1. N_2 adsorption−desorption isotherms of the carbon catalysts.

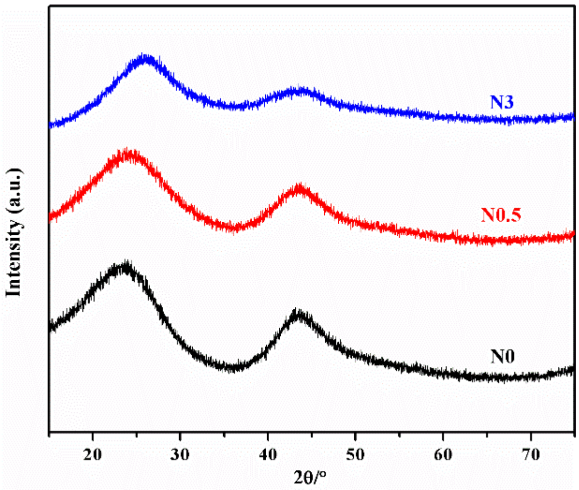

Figure S2. XRD patterns of the carbon catalysts.

The results of XRD patterns are shown in Figure S2. No characteristic reflection at 13˚ and 27˚ for C_3N_4 was detected on the XRD patterns for all the samples. only two broad peaks at 23˚ and 44˚ are present, indicating a good dispersion of carbon nitride on the surface. As the N content increase, a slightly positive shift can be observed of the broad peak at 23˚, suggesting a decrease of interlayer distances for the graphitic carbon caused by the formation of carbon nitride.[3]

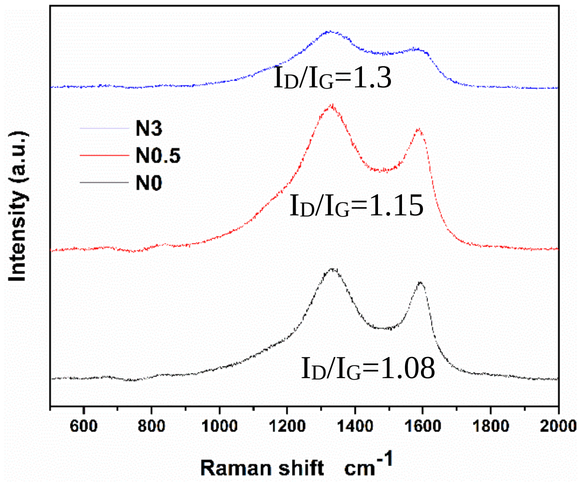

Figure S3. Raman spectra of the carbon catalysts.

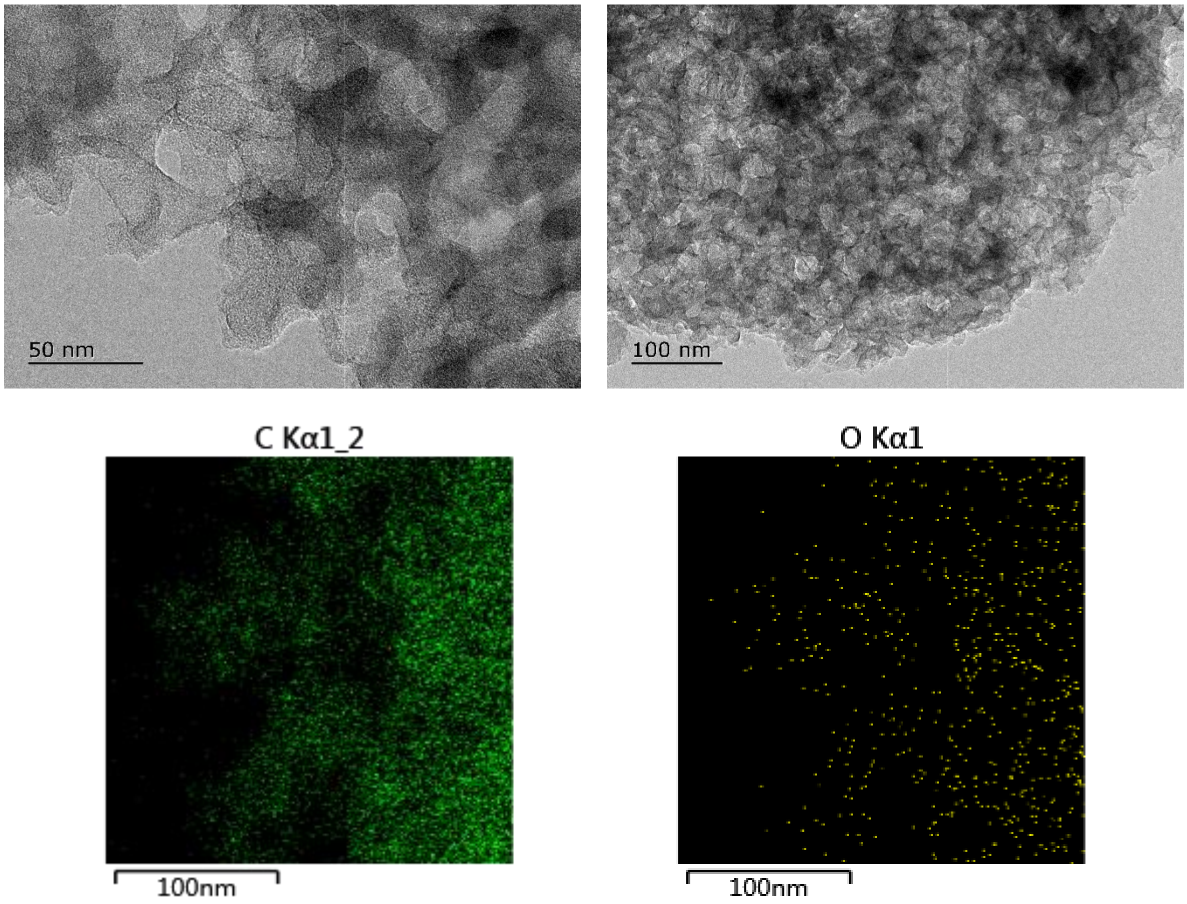

Figure S4. HR-TEM images with the corresponding elemental mapping of the N0 catalyst.

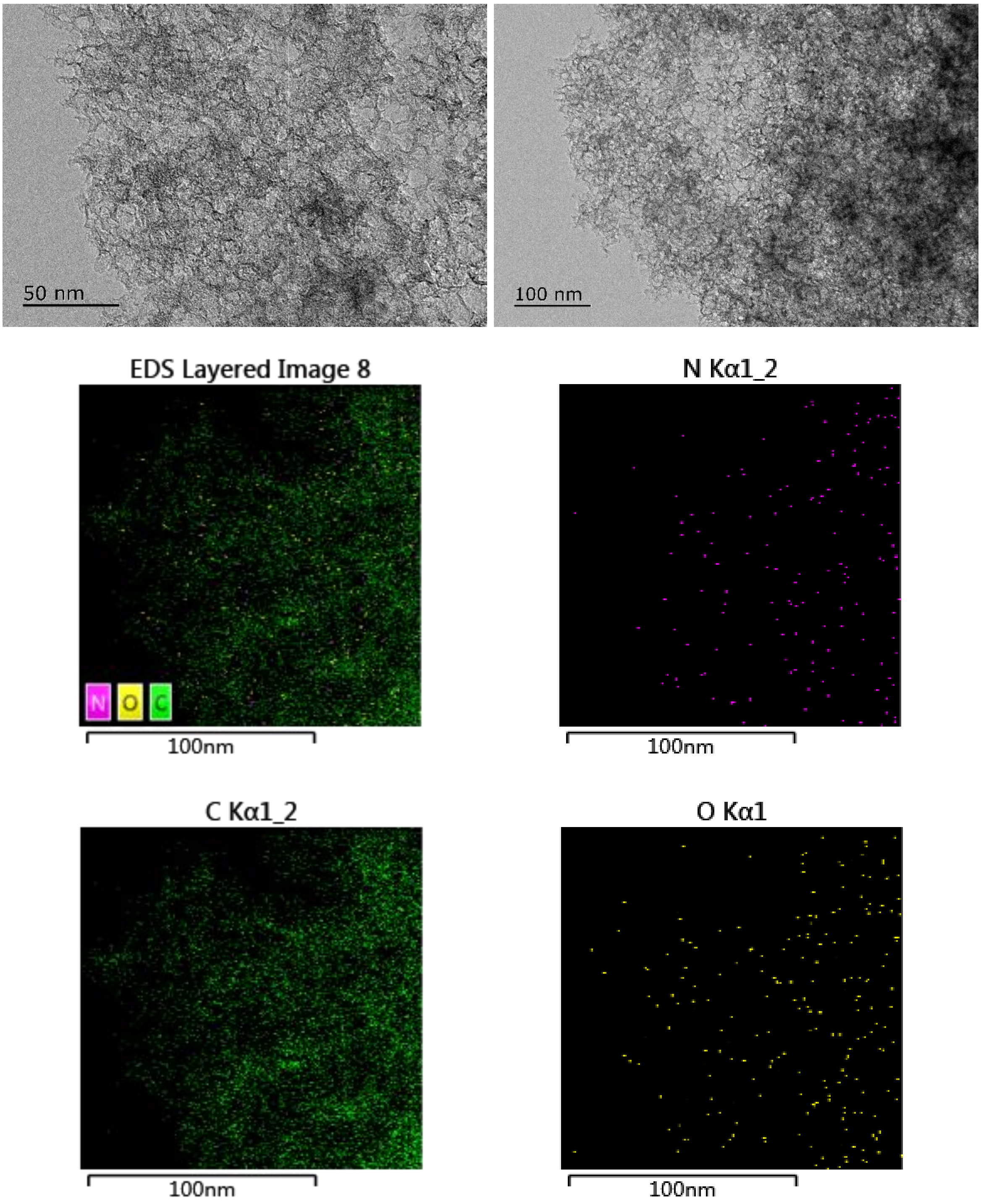

Figure S5. HR-TEM images with the corresponding elemental mapping of the N0.5 catalyst.

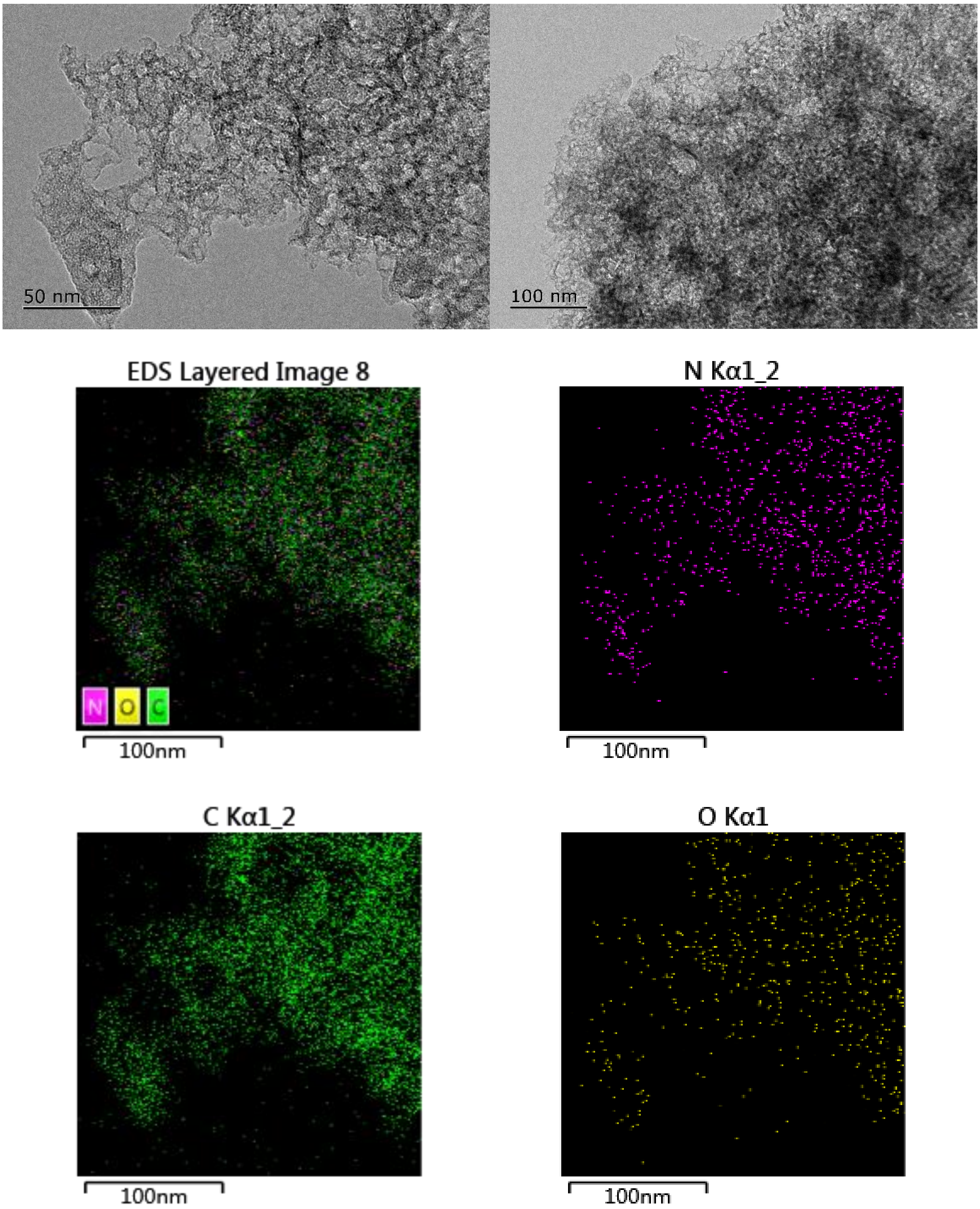

Figure S6. HR-TEM images with the corresponding elemental mapping of the N3 catalyst.

Table S2. Elements concentration in the N-doped carbon catalysts.

Catalyst	C	N	O
**N0**	94.7%	0	5.3%
**N0.5**	90.9%	4.2%	4.9%
**N3**	78.1%	15.6%	6.3%

Table S3. Relative N species distribution in the N-doped carbon catalysts*.

Catalyst	Pyridinic N	Pyrrolic N	Graphitic N
**N0**	0	0	0
**N0.5**	15.47%	81.57%	2.96%
**N3**	44.26%	25.33%	30.41%

*Typical XRS N peak and deconvolution of the peak of N3 is presented in Figure S7.

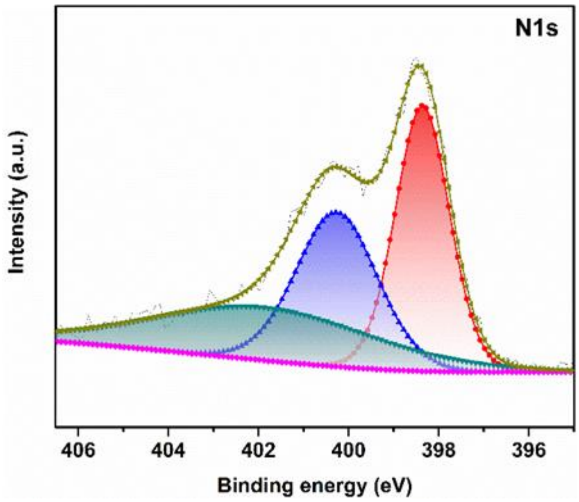

Figure S7. N1s XPS spectra of N3.

Table S4. The absolute amount of N species distribution in the N-doped carbon catalysts (g/g_{cat})

	**Pyridinic N**	**Pyrrolic N**	**Graphitic N**
**N0**	0	0	0
**N0.5**	6.5E-3	3.43E-2	1.24E-3
**N3**	6.9E-2	3.95E-2	4.74E-2

S3: Thermodynamic analysis

$C_2H_4 + 0.5O_2 + 2HCl \rightarrow C_2H_4Cl_2 + H_2O$ ΔH = -239.7 kJ/mol (1)

$C_2H_4Cl_2 \rightarrow C_2H_3Cl + HCl$ ΔH = 73.0 kJ/mol (2)

$C_2H_4 + 0.5O_2 + HCl \rightarrow C_2H_3Cl + H_2O$ ΔH = -166.7 kJ/mol (3)

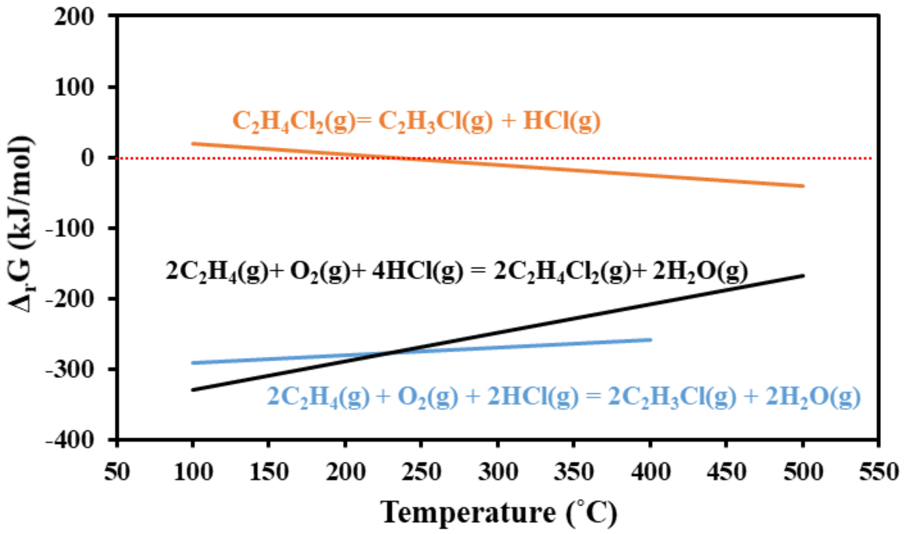

Figure S8. The Gibbs Energy (Δ_rG) as a function of temperature for the related reactions, calculated by HSC 6.0 software.

S4: Catalytic performance

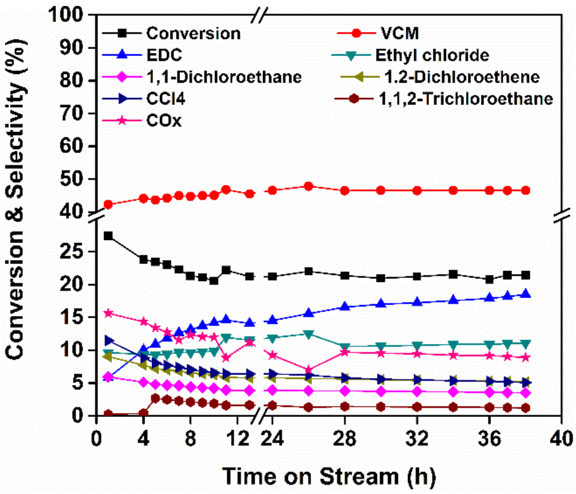

Figure S9. Catalytic results of the N0.5 catalyst for ethylene oxychlorination, Reaction condition: 250 °C, 1 bar, W_{cat}=0.8 g, gas hour space velocity: 4350 ml/h·g_{cat}, $F_{C_2H_4}$=4 ml/min,P_{total} = 1 bar, with a ratio of $C_2H_4/O_2/HCl$=2:1:2.

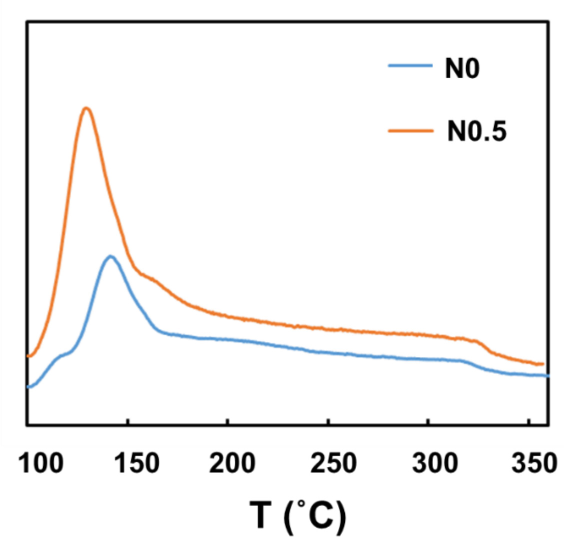

Figure S10. C_2H_4-TPD profiles on the carbon catalysts. Conditions: W_{cat} =0.5 g, F_{Ar}=50 ml/min, 10 °C/min.

Table S5. The ethylene reaction rate of ethylene oxychlorination calculated based on the N-species (pyridinic, pyrrolic, and graphitic). Reaction condition: 250 °C, W_{cat}=0.8 g, gas hour space velocity: 4350 ml/h·g_{cat}, $F_{C_2H_4} = 4$ ml/min, $P_{total} = 1$ bar, $C_2H_4/O_2/HCl$=2:1:2.

**mol/$g_{N\ specie}$s**	**Pyridinic N**	**Pyrrolic N**	**Graphitic N**
**N0.5**	1.0E-4	2.1E-5	7.0E-4
**N3**	6.0E-6	1.1E-5	8.7E-6

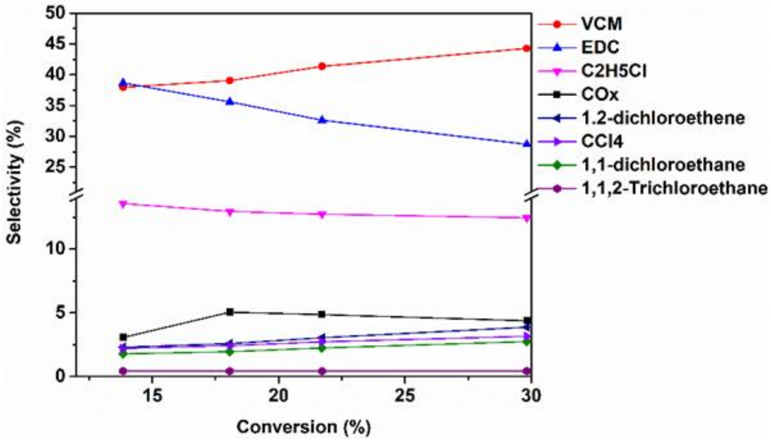

Figure S11. Product selectivity vs. ethylene conversion at 250 °C and 1 bar with the feeding molar ratio of $C_2H_4/O_2/HCl$= 2:1:2 on N0.5.

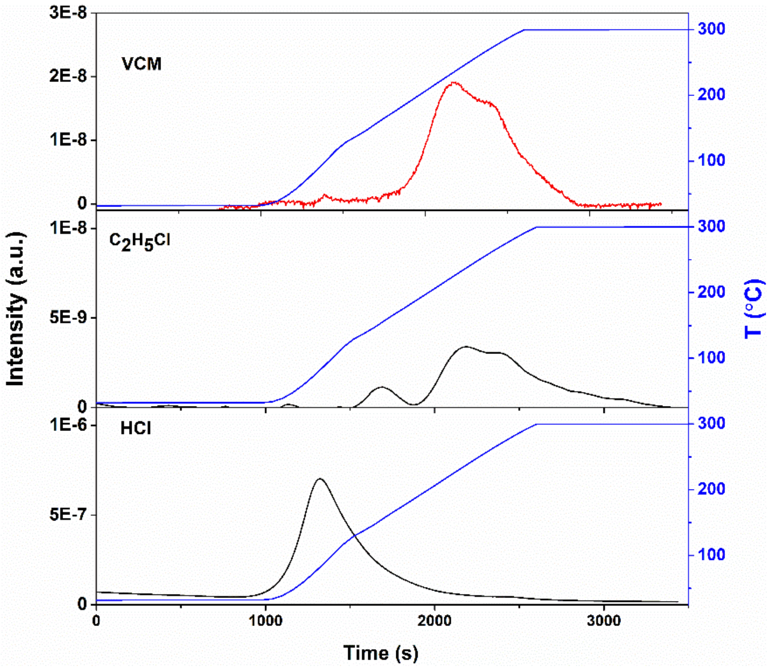

Figure S12. C_2H_4/O_2 TPSR of N0.5 after saturated adsorbing of HCl. Conditions: W_{cat}=0.5 g, 10 °C/min, F_{total}=100 ml/min, $F_{C_2H_4} = 10\ \text{ml/min}, P_{total} = 1$ bar, C_2H_4/O_2=2 diluted in Ar.

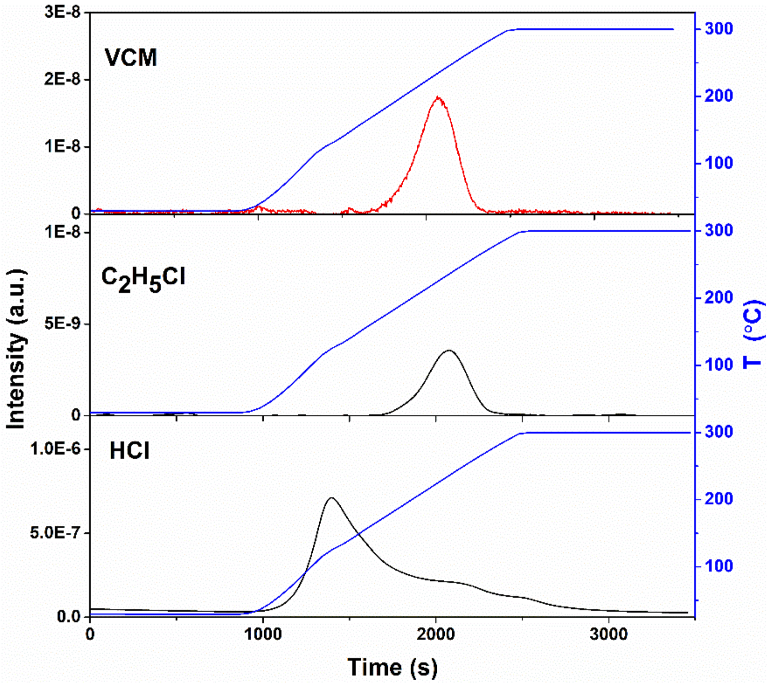

Figure S13. C_2H_4/O_2 TPSR of N3 after saturated adsorbing of HCl. Conditions: W_{cat}=0.5 g, catalyst,10 °C/min, F_{total}=100 ml/min, $F_{C_2H_4} = 10\ ml/min, P_{total} = 1$ bar, C_2H_4/O_2=2 diluted in Ar.

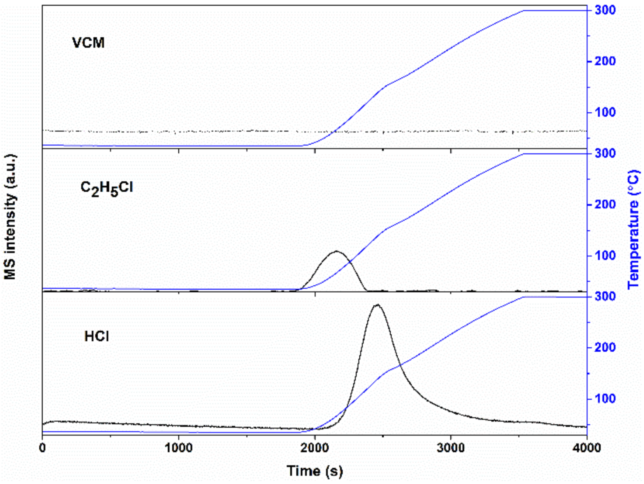

Figure S14. C_2H_4 TPSR of N0.5 after saturated adsorbing of HCl. Conditions: W_{cat}=0.5 g, catalyst,10 °C/min, F_{total}=100 ml/min, $F_{C_2H_4} = 10$ ml/min, $P_{total} = 1$ bar, diluted in Ar.

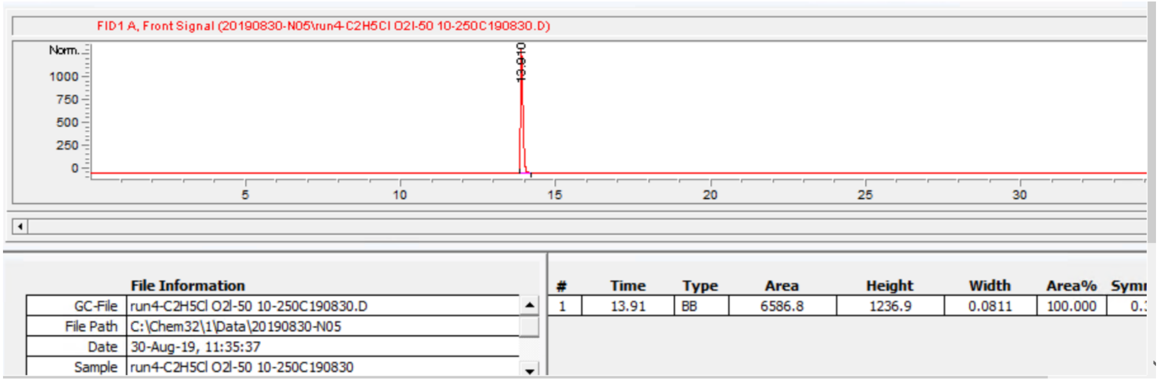

Figure S15. GC FID result of the ethyl chloride oxidation on N0.5. Reaction condition: W_{cat}= 1 g, T = 250 °C, P_{total}= 1 bar, flow rate of the reactants $F_{1\%\ C_2H_5Cl}/F_{20\%\ O_2}$: 50/10 (ml/min).

The only peak is C_2H_5Cl, no VCM, or EDC can be detected. Herein, it can be proved that there is no oxidative dehydrogenation of the C_2H_5Cl reaction occurs on the N-doped carbon catalyst.

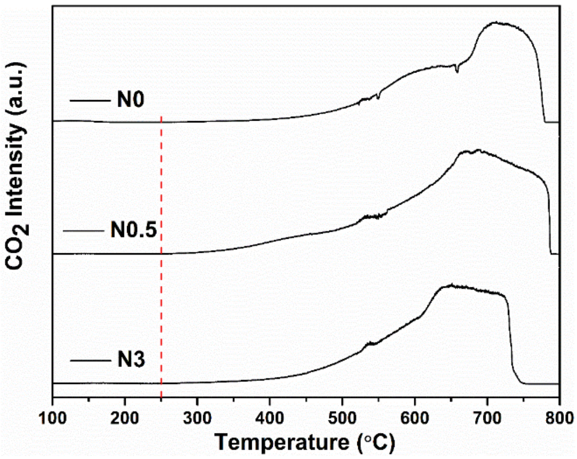

Figure S16. CO_2 formation of the carbon catalysts in O_2. Conditions: W_{cat}=0.05 g, F_{total}=50 ml/min, 6% O_2 in Ar, 5 °C/min.

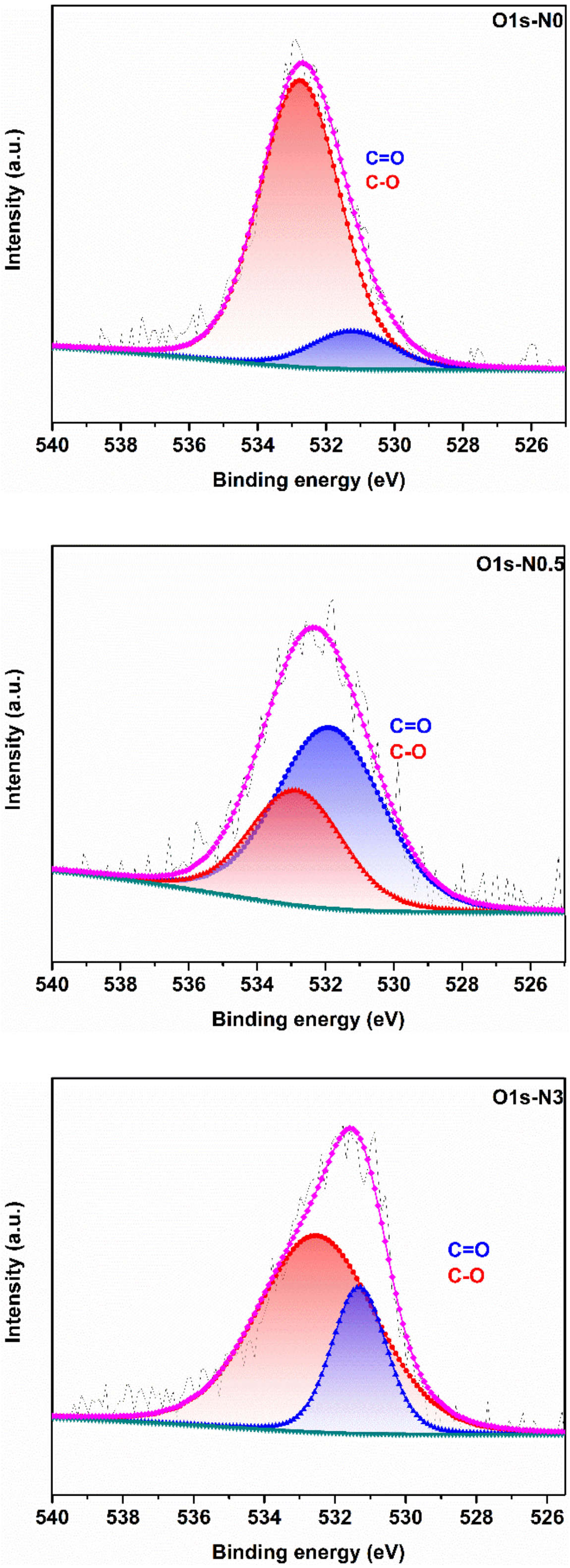

Figure S17. O1s XPS spectra of all the carbon catalysts.

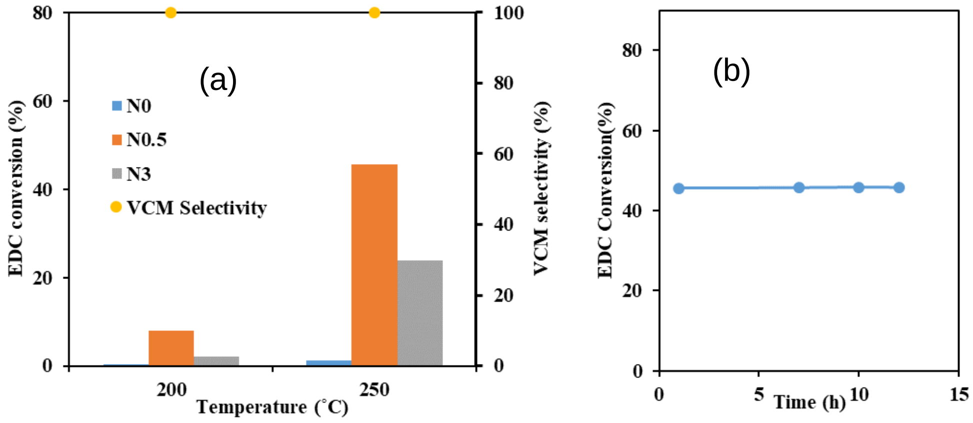

Figure S18. a) EDC conversion versus temperature in the dehydrochlorination of EDC by the bubbling method using He as the carrier gas. b) Stability test at 250 °C on N0.5 catalyst. Conditions: W_{cat}=0.5 g, F_{He}=20 ml/min, P_{EDC}=0.1 bar, $P_{total} = 1$ bar.

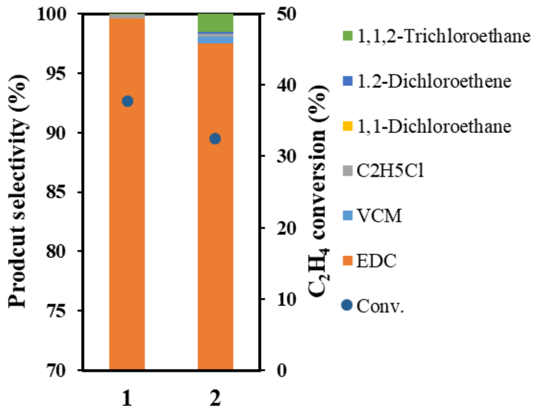

Figure S19. Catalytic results of the ethylene oxychlorination on the CeCu/Al_2O_3 catalyst. Reaction conditions: W_{cat}= 0.2 g, T=250 °C, C_2H_4/O_2/HCl= 2:1:4, gas hour space velocity: 17400 ml/h·g_{cat}, $F_{C_2H_4}$=4 ml/min. Colum 2 is adding 1000 ppm VCM inside the feeding gases.

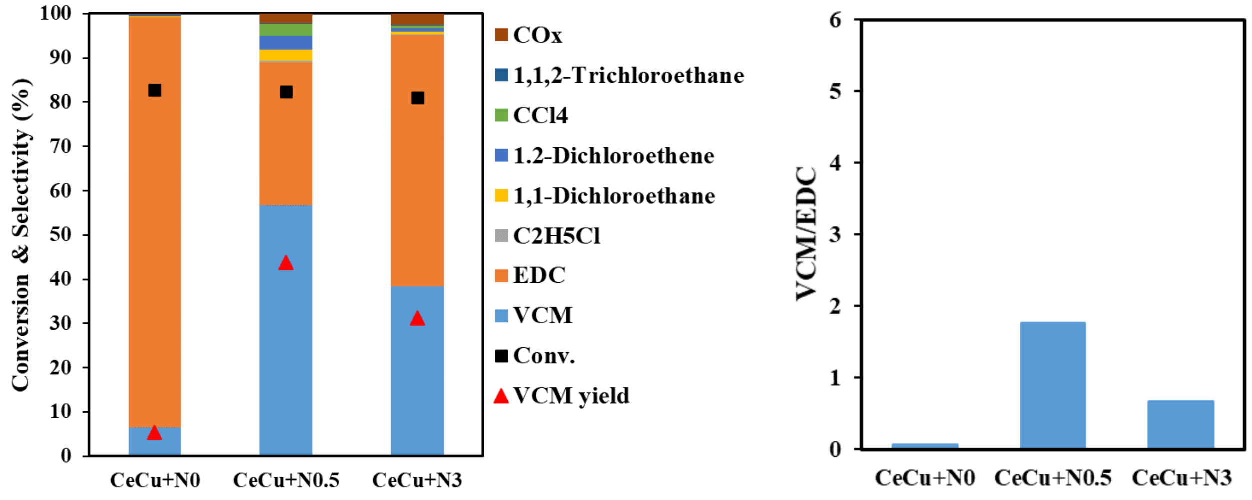

Figure S20. a) Catalytic results of the dual-bed catalyst (CeCu/Al_2O_3 at the top, N-doped carbon at the bottom), b) VCM-to-EDC ratio. Conditions: 250 °C, W_{cat}: 0.2g/0.8g, gas hour space velocity: 3480 ml/h·g_{cat}, $F_{C_2H_4}$= 4 ml/min, P_{total} = 1 bar, with the gas ratio of $C_2H_4/O_2/HCl$= 2:1:4.

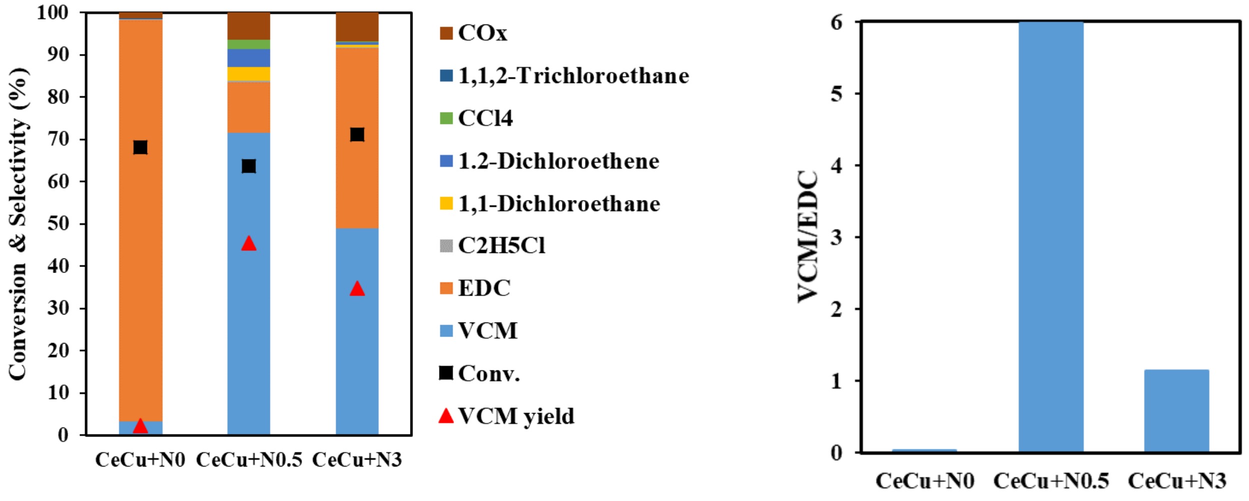

Figure S21. a) Catalytic results of the dual-bed catalyst (CeCu/Al_2O_3 at the top, N-doped carbon at the bottom), b) VCM-to-EDC ratio. Conditions: 250 °C, catalysts mass:0.2g/0.8g, gas hour space velocity: 3480 ml/h·g_{cat}, $F_{C_2H_4}$=4 ml/min, P_{total} = 1 bar, with the gas ratio of $C_2H_4/O_2/HCl$= 2:1:2.

Table S6. Catalytic results of the dual-bed method (0.5 g CeCu/Al_2O_3 at the top, 1.5 g N0.5 at the bottom) Conditions: 250 °C, gas hour space velocity: 1740 ml/h·gcat, $F_{C_2H_4}$=4 ml/min, $P_{total} = 1$ bar, $C_2H_4/O_2/HCl$=2:1:2.

**Conv./%**	**Selectivity/%**								VCM yield
	COx	VCM	EDC	C_2H_5Cl	1,1-$C_2H_4Cl_2$	1,2-$C_2H_2Cl_2$	CCl4	1,1,2-$C_2H_3Cl_3$	
94.8	3.3	79.9	11.3	0.1	2.8	0.7	1.7	0.2	75.7

S5: Reaction network and reaction mechanism

Deacon reaction

Direct Deacon reaction was performed on the N-doped carbon catalyst at 250 °C.

$$4HCl+O_2\rightarrow 2Cl_2+2H_2O$$

Cl_2 gas was analyzed by the iodometric titration. The KI (potassium iodide) solution (0.1 mol/L, 100 ml) was used for titration following the reaction

$$\mathrm{Cl_2} + 2\mathrm{KI} \rightarrow 2\mathrm{KCl} + \mathrm{I_2}$$

The formed Cl_2 will react with KI with I_2 formed, and the color of the solution will be changed. No color changes have been recorded, suggested that no Deacon reaction occurred.

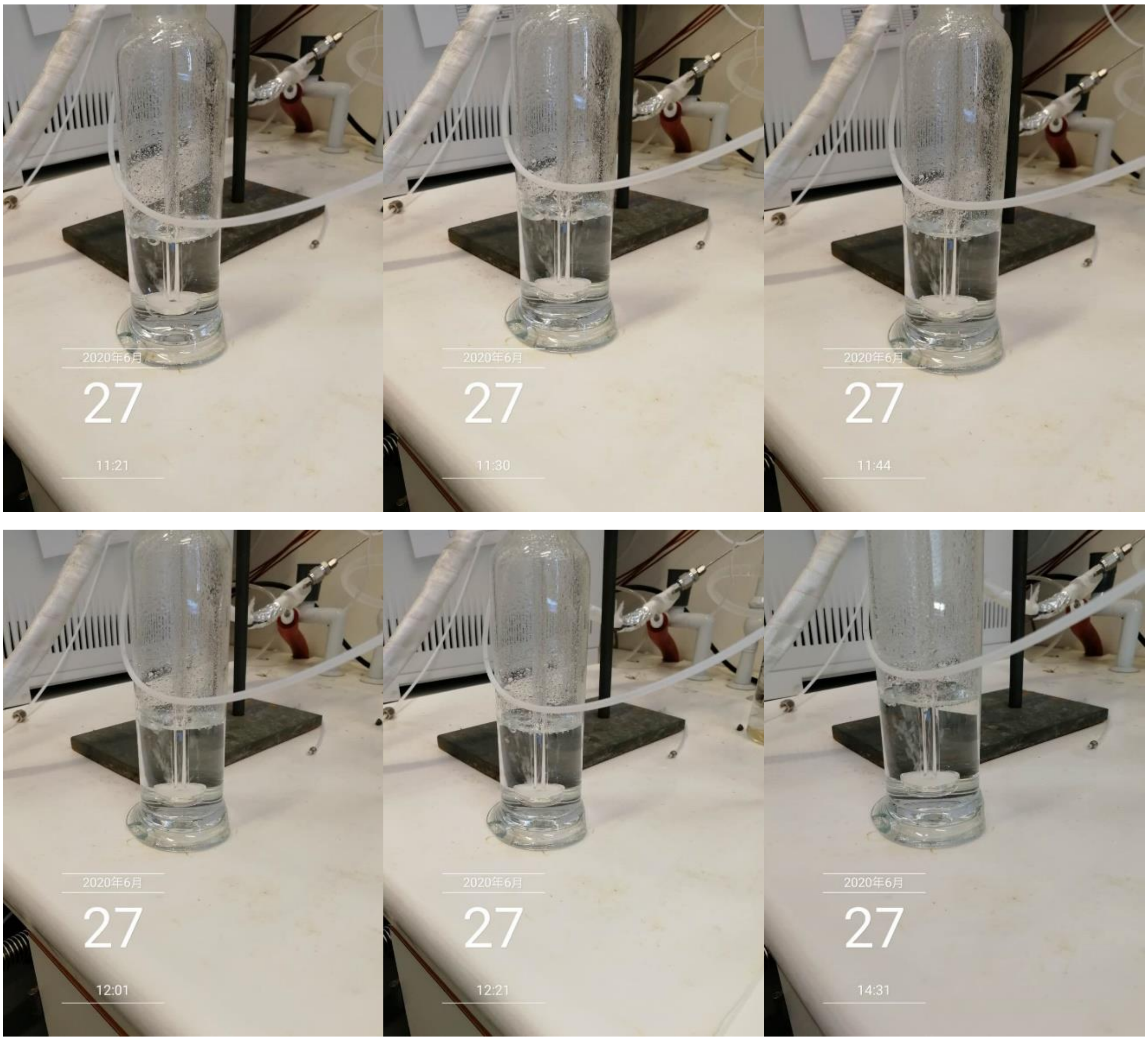

Reaction network:

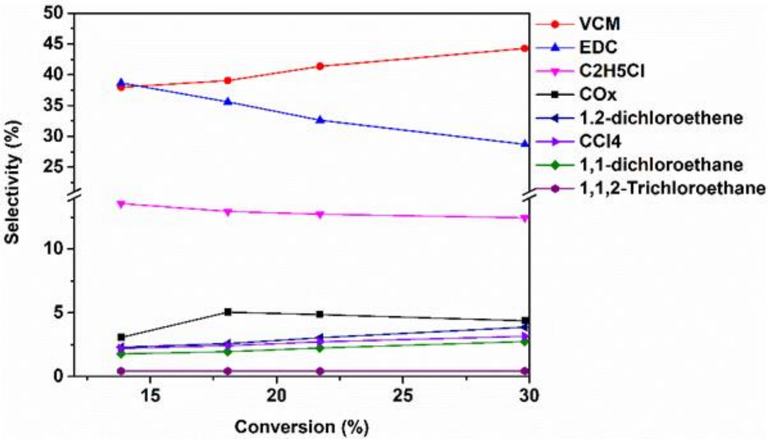

Figure S11. Product selectivity vs. ethylene conversion at 250 °C and 1 bar with the feeding molar ratio of 2:1:2 on N0.5.

With increasing conversions, the selectivity of EDC decreased and VCM selectivity increased. It suggests that EDC is an unstable primary product, while VCM is the primary product plus the secondary product from EDC dehydrochlorination. The selectivity of ethyl chloride (EC) decreased slightly with increasing conversion, suggesting the unstable primary product.

Proposed reaction mechanism of ethylene oxychlorination:

It has been well known that hydrocarbons and oxygen can be adsorbed on the carbon surfaces and it has been reviewed previously.[4]

Ethylene TPD spectra in Figure S10 indicated ethylene can be adsorbed (step R1) on the surface of both carbon and N doped carbon. N doping slightly enhanced the ethylene adsorption and the desorption occurred at a slightly low temperature, suggesting slightly weaker adsorption on N-doped carbon than the carbon surface. The π–π stacking interactions were formed between the ethylene and carbon surfaces.[4] Oxygen can also be adsorbed (R2) and dissociated (R3) on the carbon surfaces and defects.[4]

HCl−TPD spectra in Figure 3 indicated that HCl adsorbed (R4) on N sites but not on the carbon surface.

The temperature-programmed surface reaction (TPSR) of ethylene on the surface with the pre-adsorbed HCl is shown in Figure S18, only the ethyl chloride was produced (R10, R11). Both the HCl−TPD and ethylene TPSR reveals the associative adsorption of HCl (R4).

The TPSR of ethylene and oxygen on the surface with the pre-adsorbed HCl shows the formation of both the VCM and ethyl chloride in Figure S12 and S13. The adsorbed EDC seems to be directly dehydrochlorinated to VCM. Compared to ethylene TPSR, oxygen is necessary for the formation of EDC and VCM. Directly dissociation of HCl seems to be difficult, and oxygen assisted HCl dissociation (R5) seems to be essential for the activation of HCl. H in HCl reacts with OH* to form water (R7, R9).

The adsorbed Cl* preferably reacts with adsorbed ethylene to EDC (R6, R8) instead to be combined to form Cl_2 (R12). The recombination of surface Cl* seems to be not favorable and no Deacon reaction was observed.

Ethyl chloride (C_2H_5Cl) is the byproduct of the reaction. VCM can in principle be produced by oxidative dehydrogenation of ethyl chloride. The reaction of ethyl chloride and oxygen was performed on the N-doped carbon. No VCM was detected and the main product is ethylene. Oxidative dehydrochlorination instead of oxidative dehydrogenation occurred.

Based on the above experimental evidence and discussion, the reaction mechanism for direct produce VCM from ethylene oxychlorination is proposed and summarized:

C_2H_4 (g) + * = C_2H_4* R1

O_2 (g) + * = O_2*	R2
O_2* + * = 2O*	R3
HCl (g) + * = HCl*	R4
HCl* + O* = Cl* +OH*	R5
C_2H_4* +2Cl* = ClC_2H_4Cl* +*	R6
2OH* = H_2O* +O*	R7
ClC_2H_4Cl* = ClC_2H_4Cl (g) + *	R8
H_2O* =H_2O (g) + *	R9
C_2H_4* + HCl* = C_2H_5Cl* + *	R10
C_2H_5Cl* = C_2H_5Cl (g) + *	R11
Cl*+Cl*=Cl_2	R12

* is the active site.

References

[1] K. R. Rout, E. Fenes, M. F. Baidoo, R. Abdollahi, T. Fuglerud, D. Chen, *ACS Catal.* **2016**, *6*, 7030-7039.

[2] G. Liu, Q. Chen, E. Oyunkhand, S. Ding, N. Yamane, G. Yang, Y. Yoneyama, N. Tsubaki, *Carbon* **2018**, *130*, 304-314.

[3] X. Sun, X. Liu, Y. Qin, L. Qiang, Y.-P. He, D. Su, L. Song, Z. Sun, *Ind. Eng. Chem. Res.* **2019**, *58*, 5404-5413.

[4] D. Chen, A. Holmen, Z. Sui, X. Zhou, *Chin. J. Catal.* **2014,** *35*, 824-841.
